# Randomized Phase II study of the safety, efficacy and immune response of GVAX pancreas (with cyclophosphamide) and CRS-207 with or without nivolumab in patients with previously treated metastatic pancreatic adenocarcinoma (STELLAR)

**DOI:** 10.1186/2051-1426-3-S2-P155

**Published:** 2015-11-04

**Authors:** Dung T Le, Todd S Crocenzi, Jennifer N Urum, Eric R Lutz, Daniel A Laheru, Elizabeth A Sugar, Robert H Vonderheide, George A Fisher, Andrew H Ko, Aimee L Murphy, Katherine McDougall, Sandy Ferber, Dirk G Brockstedt, Elizabeth M Jaffee

**Affiliations:** 1 The Sidney Kimmel Comprehensive Cancer Center at Johns Hopkins, Baltimore, MD, USA; 2 Providence Cancer Center, Portland, OR, USA; 3 The Sidney Kimmel Comprehensive Cancer Center at Johns Hopkins, Baltimore, MD, USA; 4 Departments of Biostatistics and Epidemiology, Johns Hopkins Bloomberg School of Public Health, Baltimore, MD, USA; 5 University of Pennsylvania, Baltimore, MD, USA; 6 Stanford University School of Medicine, Stanford, CA, USA; 7 UCSF Helen Diller Family Comprehensive Cancer Center, San Francisco, CA, USA; 8 Aduro BioTech, Inc., Berkeley, CA, USA; 9 Aduro Biotech, Berkeley, CA, USA; 10 Array Biostatistics LLC, Evanston, IL, USA; 11 Aduro Biotech, Inc., Berkeley, CA, USA

## Background

A heterologous prime-boost vaccination strategy using GVAX pancreas vaccine and CRS-207 is showing promise in patients with pancreatic adenocarcinoma (PDA) (Le, JCO 2015). Furthermore, blockade of the immune checkpoint programmed death-1 (PD-1) is active in some cancers. Combinatorial strategies aimed at priming tumor antigen-specific T cells while simultaneously blocking negative checkpoints may be necessary to improve outcomes in PDA. GVAX is composed of allogeneic pancreatic cancer cells modified to express GM-CSF and induces a broad response against multiple tumor antigens. GVAX is given with low-dose cyclophosphamide (CY) to inhibit regulatory T cells. CRS-207 is live-attenuated *Listeria monocytogenes* engineered to express the tumor-associated antigen mesothelin. CRS-207 boosts responses against mesothelin and is unique in its capacity to stimulate both innate and adaptive immunity by activating T cells and NK cells. Nivolumab is an antibody against PD-1.

## Methods

This is a Phase II study comparing CY/GVAX and CRS-207 with or without nivolumab in subjects with PDA who failed only one chemotherapy regimen for metastatic disease. Subjects are randomized in a 1:1 ratio to receive either 2 doses of CY/nivolumab/GVAX and 4 doses of nivolumab/CRS-207 (Arm A) or 2 doses of CY/GVAX and 4 doses of CRS-207 (Arm B). The primary objective is to compare OS between Arms A and B. Secondary/exploratory objectives include: assessment of safety and clinical responses (tumor assessments and CA19-9 levels) and correlation of *Lm*- and mesothelin-specific T cell and other immunological responses with OS, progression-free survival and best overall response.

